# Progression, Management, and Outcome of Aortic Valve Stenosis in Systemic Sclerosis: A Case Series

**DOI:** 10.3390/jcdd11090274

**Published:** 2024-09-04

**Authors:** Andrea Vergara, Antonio Orlando, Eleonora Caiazza, Serena Vettori, Giovanna Cuomo, Paola Argiento, Emanuele Romeo, Rosa Franzese, Berardo Sarubbi, Michele D’Alto

**Affiliations:** 1 Adult Congenital Heart Disease Unit, Department of Cardiology, Monaldi Hospital, 80131 Naples, Italy; antonio.orlando1395@gmail.com (A.O.); eleonoracaiazza15@gmail.com (E.C.); paola.argiento@libero.it (P.A.); ema.romeo@alice.it (E.R.); franzeserosa07@gmail.com (R.F.); berardo.sarubbi@virgilio.it (B.S.); 2 Internal Medicine, Department of Medicine, Monaldi Hospital, 80131 Naples, Italy; serenavettori05@gmail.com; 3 Department of Precision Medicine, University of Campania “Luigi Vanvitelli”, 80138 Naples, Italy; giovanna.cuomo@unicampania.it

**Keywords:** aortic valve stenosis, systemic sclerosis, connective tissue disease

## Abstract

Background: In systemic sclerosis (SSc), cardiac involvement is frequent, heterogeneous, and related to a poor prognosis. Due to a longer life expectancy, the development of degenerative aortic stenosis (AS) is not uncommon. The aim of this article is to report the characteristics of AS in SSc, analyzing the rate of progression, the management, and the outcome. Methods: This is a case series conducted at the Department of Cardiology of Monaldi Hospital, Naples, Italy. Results: From January 2007 to December 2022, we analyzed 234 patients with SSc. Ten/234 patients (4.3%) showed severe AS and were included in the analysis (age 75.5 years [IQR 58–84], nine females). Nine had limited and one diffuse SSc. Two patients were in NHYA/WHO II and eight in NYHA/WHO III. All had degenerative three-leaflet AS. Two patients showed severe AS at the first evaluation, and eight developed severe AS during the follow-up, with a time progression from moderate to severe AS of 3.2 ± 1.1 years (progression rate −0.190 ± 0.012 cm^2^/year for aortic valve area, 8.6 ± 6.1 mmHg/year for mean aortic gradient, 16 ± 7 mmHg/year for peak aortic gradient, and 0.5 ± 0.3 m/s/year for aortic peak velocity). Seven out of 10 patients underwent transcatheter aortic valve implantation (TAVI), one underwent surgical aortic valve replacement (SAVR), one was left untreated, and one was on a waiting list for TAVI. No major complications after TAVI or SAVR occurred. At a mean follow-up of 5.9 ± 3.9 years, eight patients are alive and two died. Conclusion: Severe AS is a relevant cardiac complication of SSc and must be considered in the screening and during the follow-up. Its rapid progression rate may tentatively be due to autoimmunity, degenerative burden, and chronic inflammation.

## 1. Introduction

Systemic sclerosis (SSc) is a multisystem autoimmune disease of unknown etiology; it is characterized by immune-system dysregulation, proliferative and obliterative microvascular changes, and aberrant extracellular matrix deposition ensuing in the fibrosis of multiple organs: skin, gastrointestinal tract, lungs, heart, muscle, joints, tendons, and kidneys [1].

SSc patients are divided into two clinical subsets, based on the extent of skin involvement: Limited cutaneous SSc (lcSSc), in which skin thickening is distal to knees and/or elbows; diffuse cutaneous SSc (dcSSc), in which skin thickening of the limbs is proximal to the trunk or involves the trunk. Face skin thickening can be present in both subsets. LcSSc patients usually show an indolent disease course and a better prognosis, whereas dcSSc patients show a more aggressive disease course with early fibrotic internal organ involvement. Some patients do not show any skin involvement at all. They are called “sine scleroderma”, and are included in the lcSSc subset, as they show the same indolent course [2]. SSc patients are also classified based on the autoantibody pattern, as the presence of anticentromere antibodies (ACA) is usually associated with a more favorable outcome, and antitopoisomerase antibodies (ATA) to a more rapidly progressive disease, independently of the extent of skin involvement [1].

**Figure 1:**
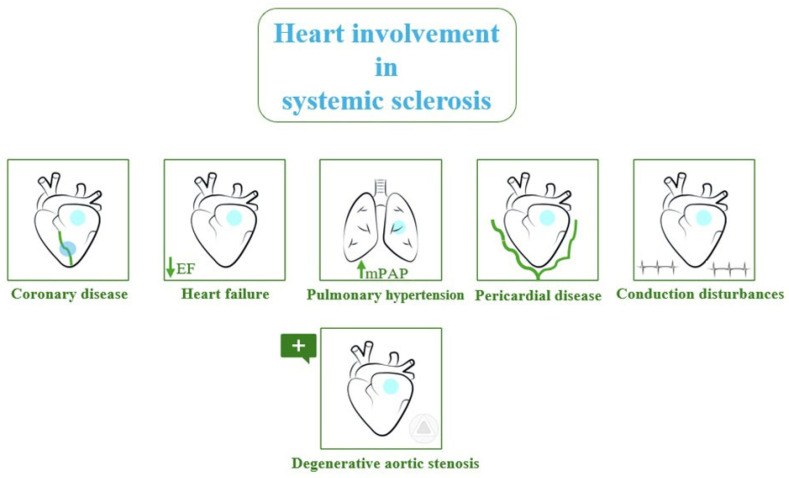
Cardiac involvement in systemic sclerosis.

The cardiac involvement is heterogeneous: ventricular dysfunction, heart failure, coronary artery disease, arrhythmias, pericardial disease, valve diseases, and pulmonary hypertension (PH) (Figure 1) [3].

It is estimated that in more than 70% of patients with SSc, there is subclinical cardiac involvement due to the presence of microvascular disease and focal interstitial fibrosis with preserved ventricular systolic function and dimension. In these cases, the diagnosis is more insidious, and suspicion arises from increased troponin and N-terminal prohormone of brain natriuretic peptide (NT-proBNP) [4].

The association between SSc and myocardial injury is common, ranging from 15 to 35%, and, more importantly, it is associated with a poor prognosis, with mortality as high as 70% at five years [5,6,7].

Most recently, attention has been paid to valve disease in SSc, which appears to be more frequent than expected [8]. 

Due to a longer life expectancy related to earlier diagnosis and better holistic management of the disease, the development of degenerative aortic stenosis (AS) has been reported in different case series [9,10].

The aim of this retrospective study was to report a case series of severe AS in SSc patients, analyzing the rate of progression, the management, and the outcome.

## 2. Detailed Case Description

The whole SSc cohort was 234 patients with a mean age of 50 years (18–79). Ten patients showed severe AS and were included in the analysis (age 75.5 (58–84) years, nine females, one male, median disease duration 20 (6–27) years). Eight patients had lcSSC and two dcSSc. Seven patients had interstitial lung disease, and seven, gastrointestinal tract involvement; none had kidney disease or digital ulcers, as shown in Table 1. 

Six patients had an active nailfold videocapillaroscopy pattern, three a late pattern; one patient had no capillaroscopy data.

Regarding the autoantibody subset, three patients were ACA positive, four were ATA positive, and three patients did not show any specific antinuclear antibody (ANA).

Two patients were in NHYA/WHO functional class II, and eight were in NYHA/WHO functional class III.

All patients presented with degenerative tri-leaflet aortic valve disease.

Two patients showed severe AS at the first echocardiographic evaluation, and eight developed severe AS during the follow-up, starting from a moderate disease. In these eight patients, the time of progression from moderate to severe AS was 3.2 ± 1.1 years. The progression rate was −0.190 ± 0.012 cm^2^/year for aortic valve area (AVA), 8.6 ± 6.1 mmHg/year for mean aortic gradient, 16 ± 7 mmHg/year for peak aortic gradient, and 0.5 ± 0.3 m/s/year for aortic peak velocity. One patient had paradoxical low flow, low gradient aortic stenosis, and the severity was confirmed by CT with a calcium score study (3400 Agatston Unit). The main echocardiographic data are summarized in Table 2.

Seven out of 10 patients underwent transcatheter aortic valve implantation (TAVI), one underwent surgical aortic valve replacement (SAVR), one was left untreated (procedure excluded futility), and one is on the waiting list for TAVI.

No patient showed major complications or sequelae after TAVI or surgery.

At a mean follow-up of 5.9 ± 3.9 years, eight are alive, and two died (one left untreated and one 12 months after TAVI).

## 3. Materials and Methods

This is a single-center case series conducted at the Department of Cardiology of the Monaldi Hospital, Naples, Italy. From November 2007 to February 2021, 234 patients with SSc were visited in our hospital. The presence of SSc was diagnosed by experienced rheumatologists, and all patients met the 2013 EULAR/ACR criteria [11]. Furthermore, all patients underwent full clinical–serological and nailfold capillaroscopy assessment.

The transthoracic echocardiogram study was performed according to the international recommendations using Vivid E9 (GE Healthcare, Milwaukee, WI, USA) with a 3.5 MHz sector probe [12]. All images in cine-loop format were analyzed offline. Severe AS is defined as follows: AVA < 1 cm^2^, indexed AVA < 0.6 cm^2^, DP > 40 mmHg, Vmax > 4 m/s, and DVI < 0.25 [13].

## 4. Discussion

The main findings of this case-series study are the following: (1) The prevalence of severe AS in SSc was 4.3%; (2) the progression rate was −0.190 ± 0.012 cm^2^/year for aortic valve area (AVA), 8.6 ± 6.1 mmHg/year for mean aortic gradient, 16 ± 7 mmHg/year for peak aortic gradient, and 0.5 ± 0.3 m/s/year for aortic peak velocity (faster than in the general population); and (3) the transcatheter and surgical approaches are valuable options with few complications.

Studies on valve disease in SSc are lacking, as primary SSc-related heart involvement has been mainly focused on the sequelae of small intra-myocardial artery vasculopathy and subsequent interstitial fibrosis, according to autopsy study findings [14]. However, valve involvement in SSc has been described since the 1980s, even though rarely reported [15]. Actually, there is no standardized definition of primary heart involvement in SSc because this largely depends on the investigating tools that have been applied in different studies [16]. Due to the prominent role acquired by advanced echocardiography methods and to the increased long-term survival of SSc patients, valve disease is increasingly being detected, with AS being the rarest occurrence [9,17,18].

### 4.1. Pathogenesis

Even though the exact pathogenesis of AS in SSc remains unclear, several potential pathogenic mechanisms might be involved, including endothelial dysfunction, inflammation, aberrant adaptive immune response and pathogenic autoantibody production, accelerated atherosclerosis, ectopic calcification deposits, and chronic hemodynamic stress associated with coexisting PH [1,19,20].

Endothelial dysfunction, due to different triggers, plays a major role in SSc. It is associated with an increased expression of adhesion molecules by endothelial cells, which induces migration of immuno-inflammatory cells in the injured tissue, vasoactive mediators’ imbalance, and acquisition of a prothrombotic phenotype, finally leading to extracellular matrix expansion and fibrosis [21]. These phenomena cause a cascade of events in which multiple players are involved, including adaptive immune system activation.

In fact, an aberrant antigen-driven immune response is observed in SSc, along with the endothelial cell modifications. This also occurs in other chronic inflammatory rheumatic and non-rheumatic diseases, including atherosclerosis and AS [1,22,23].

Mazzone A. et al. reported activated inflammatory infiltrates, neovessels, and over-expression of adhesion molecules in calcium nodules of non-rheumatic stenotic aortic valves from 26 patients undergoing surgical valve replacement [24].

Interestingly, in 2003, a study by Poggianti E. et al. correlated systemic endothelial dysfunction as assessed by flow-mediated dilation with AS, thus suggesting that AS could be an expression of atherosclerotic disease and that endothelial valve modifications could be similar to those occurring in SSc vasculopathy [25]. 

Obviously, the activation of adaptive immune response driven by (unknown) antigen-stimulated T cells also induces B-cell activation and plasma-cell differentiation with autoantibody production. Modified endothelial cells may induce anti-endothelial antibody (AECA) generation. Interestingly, AECA has been described both in SSc patients and patients with a variety of valve defects secondary to rheumatic heart disease, including AS [20,26]. Lastly, as calcium deposits are the hallmark of degenerative AS, it is likely that this mechanism can be accelerated in SSc due to both accelerated atherosclerosis and the predisposition of a distinct subset of SSc patients (i.e., patients with limited skin sclerosis and indolent disease course, meaning longer survival) to accumulate calcium deposits in the interstitium of injured tissues [27]. 

In regard to acute systemic inflammation, this is rarely clinically detectable in SSc. However, around 25% of a large SSc cohort have been found with increased serum levels of C-reactive protein (CRP) [28], and CRP serum levels have been associated with unfavorable outcomes in SSc patients with cardiac involvement, including PH [29].

Data about the predictive role of CRP in AS or any valve disease in SSc have never been explored; nevertheless, data about the predictive role of CRP in calcific aortic valve disease or AS in the general population remain controversial [30,31]. 

In addition, in an open-label retrospective study, PH has been associated with AS in 21% of SSc cases, showing progression to severe AS in around one-third of them [32].

Along with apparent shared pathogenic mechanisms, the association of SSc and AS could also be favored by demographic factors and other SSc-related clinical features, like age, gender, and disease subset (limited or diffuse skin involvement). In fact, the majority of the AS–SSc cases described in the literature, including in our series, are 70-year-old or older females who have a limited cutaneous disease with a more indolent disease course and a longer survival [33].

It is well-known that AS occurs later in females than in males. It is, therefore, possible that, taken together, these factors contribute to increased observation of AS cases in SSc.

Despite the pathophysiological mechanism of AS in SSc remaining unknown, the age, the autoimmunity, the degenerative burden, and the chronic inflammatory status might accelerate the progression of AS in these patients [34,35].

### 4.2. Aortic Stenosis Prevalence and Progression

The prevalence of AS varies with age and comorbidities. From a study of 164 patients, prevalence values were 0.2% in the 50–59-year group, 1.3% in the 60–69-year group, 3.9% in the 70–79-year group, and 9.8% in the 80–89-year group [36].

Similar results emerge from a meta-analysis of 9723 patients aged > 75 years: the prevalence of AS was 12.4%, while the prevalence of severe AS was 3.4% [37].

In our SSc population, although limited in number, the prevalence is 4.3%, with a mean age of about 50 years and a wide age range. These findings suggest a higher prevalence and an earlier onset of AS in SSc patients. There is still little data in the literature, and further larger multicenter studies are needed to confirm these findings.

Several studies have evaluated the progression of AS in the general population. A previous echocardiographic investigation has shown an annual increase of the mean trans-aortic pressure gradient by 6.3 to 8.3 mmHg and an annual decrease of the aortic valve opening area by 0.14 cm^2^ [35]. 

Piper, in a series of 257 patients with AS, observed that the aortic valve obstruction progression increases not linearly but exponentially, according to the degree of valve calcification [38]. 

These authors conclude that if the aortic valve area and the degree of calcification are known, the hemodynamic progression of the lesion may be predictable. Nevertheless, the intervals of confidence are wide, indicating large inter-individual differences and the impossibility of forecasting the progression rate in an individual patient. 

Kebed et al. analyzed 916 patients with mild to severe AS and followed with echocardiographic examination. In this study, the rate of progression of AS was faster in males and white patients than in blacks and other ethnicities. The overall rate of AS progression by change in AVA was −0.070 ± 0.003 cm^2^/year. There was no difference in the progression of the valve area according to age. Furthermore, there was an inverse relationship between the initial degree of severity of AS and progression, probably because mild forms of AS rarely progress to forms beyond mild. Left ventricular systolic function does not influence the rate of AS progression [39]. 

In a meta-analysis of 24 studies that included 5450 patients, Willner observed that the progression of AS is faster when severity at baseline is greater: 0.07 cm^2^/y in mild AS, 0.08 cm^2^/y in moderate AS, 0.09 cm^2^/y in severe AS; therefore, there is a strong correlation between the severity of AS at baseline and the rate of anatomic and hemodynamic valve disease progression [40].

In our small case series, the progression rate was about three times faster than in Kebed’s series (−0.190 ± 0.012 cm^2^/year vs. −0.070 ± 0.003 cm^2^/year), suggesting an important role for the underlined autoimmune disease as a possible accelerating factor of AS progression. 

Early identification of severe AS is mandatory. In fact, if left untreated, AS might increase the side effects of dihydropyridine calcium channel blockers, commonly used in SSc, or preclude the use of the standard therapies for pulmonary artery hypertension (PAH).

### 4.3. Treatment and Outcomes

Frailty, common in SSc, is also an important factor that can significantly affect aortic valve treatment outcomes. Multiple comorbidities presenting in SSc patients make the Society of Thoracic Surgeons (STS) score and logistic EuroSCORE suboptimal models for an adequate risk stratification [41]. 

SSc patients with severe AS are likely frail, elderly women. The frailty and the possible multiple medical comorbidities must be taken into account to depict the futility of TAVI or surgical aortic valve replacement (SAVR), defined as the combination of death and/or the absence of clinical improvement at follow-up [34,42,43,44]. 

TAVI shows potential advantages over SAVR in SSc patients: The avoidance of general anesthesia, sternotomy on a difficult chest anatomy, a shorter in-hospital stay, and an easier periprocedural management. On the other hand, patients with SSc may develop intraprocedural vascular complications due to vascular fragility and small-caliber vessels. Previous observations confirm that TAVI is a successful procedure with favorable long-term clinical outcomes in SSc patients [45]. 

Nevertheless, operators should consider possible challenges due to the smaller vessel size caliber, the vessel tortuosity, the endothelial disease inducing high rates of macro-microvascular involvement, the possible kidney damage, the age, and the small body size (often the patients are old women). The contrast-related acute kidney injury should be prevented with appropriate hydration protocols. Finally, because conduction disturbances are very common in SSc patients, the increased risk of high atrioventricular block must be carefully evaluated in the immediate post-procedural period. However, multidisciplinary evaluation is necessary to decide the best strategy case-by-case [35]. 

## 5. Conclusions

Aortic valve stenosis is one of the possible manifestations of cardiac involvement in patients with SSc; therefore, a careful echocardiographic evaluation of the aortic valve is required during follow-up. The rate of progression of AS is more rapid in patients with SSc compared to the general population. Nevertheless, there are no models yet to predict the rate of progression of AS, probably because multiple mechanisms contribute to it: autoimmunity, degenerative burden, and chronic inflammation. The therapeutic approach to AS in SSc patients can be percutaneous or with traditional surgery. The choice of approach is multifactorial and depends mainly on the patient’s frailty, anatomical features, and comorbidities.

## Figures and Tables

**Table 1:** Demographic, clinical, serological, and capillaroscopy features of the 10 enrolled systemic sclerosis patients. SSc = systemic sclerosis; Ab = autoantibody subset; DD = disease duration in years (y); ILD = interstitial lung disease; GIT = gastrointestinal tract involvement; DU = digital ulcers; ESR = erythrocyte sedimentation rate; CRP = C-reactive protein; NVC = nailfold videocapillaroscopy; F = female; lcSSc = limited cutaneous SSc; ANA = antinuclear antibodies (e.g., patients negative for SSc marker autoantibodies other than ACA and ATA at baseline); ATA = anti-topoisomerase antibodies; lcSSc° = sine scleroderma, included in the lcSSc subset; M = male; dcSSc = diffuse cutaneous SSc; ACA = anticentromere antibodies; “−” = negative; “+” = positive.

Patients	Age	Sex	Subset	Ab	DD (y)	ILD	GIT	Kidney	DU	Calcinosis	ESR/CRP	NVC
1	84	F	lcSSc	ANA	22	−	+	−	−	−	−	late
2	76	F	lcSSc	ATA	6	+	+	−	−	−	−	active
3	75	F	lcSSc°	ATA	14	−	−	−	−	−	+	−
4	82	M	dcSSc	ANA	23	+	+	−	−	−	−	late
5	70	F	lcSSc	ATA	20	+	+	−	−	−	−	active
6	80	F	lcSSc	ACA	27	+	+	−	−	+	+	late
7	58	F	lcSSc°	ACA	20	−	+	−	−	−	−	active
8	71	F	lcSSc	ATA	20	+	−	−	−	−	−	active
9	77	F	lcSSc	ACA	19	+	+	−	−	−	−	active
10	71	F	dcSSc	ANA	18	+	−	−	−	−	−	active

**Table 2:** Echocardiographic features of patients with severe aortic stenosis and systemic sclerosis. AVA = aortic valve area; DVI = Doppler velocity index; LVEF = left ventricular ejection fraction; IVC = inferior vena cava; IVSd = interventricular septum diameter; LAVi = left atrial volume index; LVEDd = left ventricular end-diastolic diameter; LVESd = left ventricular end-systolic diameter; LVOT VTI = left ventricular outflow tract velocity time integral; TAPSE = tricuspid annular plane systolic excursion.

	Patients with Severe Aortic Stenosis and Systemic Sclerosis									
N	1	2	3	4	5	6	7	8	9	10
LVEDd (mm)	49	40	57	51	63	46	48	51	51	51
LVESd (mm)	32	22	44	42	51	29	25	42	40	41
IVSd (mm)	12	12	14	15	12	11	13	12	13	12
LVEF (%)	57	60	55	50	40	55	60	55	55	60
E/e’	4.7	12	11	15	9	9	9	11	9	20
LAVi (mL/m^2^)	31	36	37	50	37	37	38	39	42	35
VTI LVOT (cm)	10	12	12	10	7	10	12	10	11	10
Aortic Vmax (m/s)	4	4.1	4.2	4.1	4	4.1	4.1	4.4	4.9	3.7
Mean aortic gradient (mmHg)	40	48	50	45	40	42	41	48	60	35
AVA (cm^2^)	0.5	0.7	0.8	0.4	0.5	0.5	0.7	0.6	0.4	0.5
DVI	0.23	0.18	0.18	0.20	0.22	0.21	0.21	0.19	0.15	0.23
RVD1 (mm)	54	55	33	34	40	36	34	35	38	40
TAPSE (mm)	17	19	17	19	22	18	21	19	23	18
S’ wave (cm/s)	9	10	9	15	12	10	11	10	13	10
Pulmonary acceleration time (ms)	104	100	83	86	98	106	115	95	89	143
IVC (mm)	14	16	18	20	16	13	13	15	12	18
IVC inspiratory collapse (%)	55	60	60	50	60	70	65	60	55	60

## Data Availability

The data presented in this study are available upon reasonable request from the corresponding author.
